# Multiple visual objects are sampled sequentially

**DOI:** 10.1371/journal.pbio.2003230

**Published:** 2017-07-24

**Authors:** Ole Jensen, Marlies E. Vissers

**Affiliations:** Centre for Human Brain Health, School of Psychology, University of Birmingham, Birmingham, United Kingdom

## Abstract

When acting in a complex visual environment, it is essential to be able to flexibly allocate attention to parts of the visual scene that may contain goal-relevant information. The paper by Jia et al. provides novel evidence that our brains sequentially sample different objects in a visual scene. The results were obtained using “temporal response functions,” in which unique electroencephalographic (EEG) signals corresponding to the processing of 2 continuously presented objects were isolated in an object-specific way. These response functions were dominated by 10-Hz alpha-band activity. Crucially, the different objects were sequentially sampled at a rate of about 2 Hz. These findings provide important neurophysiological insights into how our visual system operates in complex environments.

## How are multiple objects in a visual scene sampled?

Given that our visual system receives a constant flow of information, mechanisms need to be in place to help prioritize relevant from less relevant visual objects [1]. Such a mechanism involves the allocation of spatial attention. Spatial attention was initially explained by a searchlight analogy [2–4]. According to this analogy, the allocation of spatial attention results in a gain increase for attended objects and a reduced gain for unattended objects. While this analogy has received strong experimental support [5–8], it may be insufficient to fully describe the spatiotemporal dynamics of attention in daily life. For instance, we usually do not attend to a single object in a visual scene for long but rather continuously explore different parts of a visual scene by shifting attention. Shifts in attention might involve either the reallocation of spatial attention (covert attention) while maintaining one’s gaze at a given location or might involve saccades (changes in the position of the eyes, i.e., overt shifts of attention).

Importantly, saccades typically occur to informative parts of the visual scene [9]. This poses an interesting conundrum: how does our visual system know how to direct our gaze to objects that have not yet been consciously perceived? One potential explanation is that even when we focus on one object in a visual scene, possible candidates for future saccades are also processed, at least partially. This scheme implies that the focus of attention is not limited to a single object or location and raises the important question of whether attention reflects a parallel or serial process [10]. The work by Jia et al. published in *PLOS Biology* provides support for serial processing of multiple objects [11].

## Identifying the dynamics of visual attention using temporal response functions

The experiments by Jia et al. were done using electroencephalogram (EEG) in combination with a technique based on so-called temporal response functions (TRF; Lalor, Pearlmutter, Reilly, McDarby, and Foxe, 2006). The basic principle underlying TFRs is that an object is presented as a flicker, of which the luminance is varied randomly over time, while the participant’s EEG is recorded. The TRF reflects the impulse response of the visual input (luminance changes of the object in the study by Jia et al.) in the brain as measured with the EEG and thus reflects the signal that best accounts for the measured EEG when convolved to the visual input [12,13]. The group of VanRullen and Macdonald previously demonstrated that the TRF of a visual stimulus is a decaying oscillatory response of about 10 Hz, which they termed perceptual echoes [13]. Importantly, the alpha-band response in the TRF links the oscillatory alpha dynamics associated with attentional modulations of sensory processing [14–16] to object-specific attentional modulation of neural processing.

**Fig 1:**
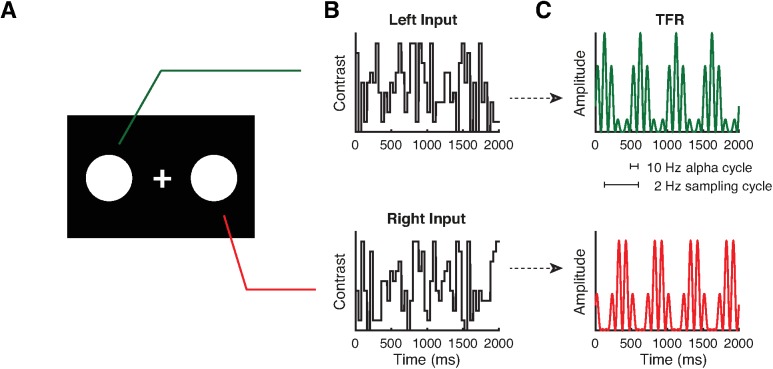
(A) In the study by Jia et al., two objects were presented. Participants were asked to detect the random appearance of a small target square in one of the objects. (B) The contrast of each object changed randomly over time. (C) The temporal response functions (TRFs) were calculated by relating the recorded EEG to the visual input train (contrast change of each object). The TRF can be considered to reflect the impulse response function that best explains the EEG when convolved with the stimulus train, also termed as perceptual echoes [13]. The TRFs were dominated by approximately 10-Hz alpha-band activity. The sequential sampling was observed as a rhythmic modulation (about 2 Hz) of the envelope of the alpha-band response.

The group of Jia et al. used TRFs to simultaneously tag two objects that were presented in the left and right hemifield with orthogonal flicker signals (Fig 1A and 1B). The time-frequency representations of the impulse responses (TRFs) for each of the stimuli were then derived from the EEG. This approach allowed for isolating the brain dynamics associated with the processing of each object, providing a measure that can be used to assess how spatial attention affects object-specific neural processing.

The first experimental observation was that the frequency content of the TRF of both objects was dominated by an approximately 10-Hz response (see Fig 2A in Jia et al.), thus reproducing the perceptual echoes of VanRullen et al. [13]. Importantly, this response was modulated by spatial attention. When one object was clearly task relevant (indicated by a 100% valid attentional cue, rendering the other object task irrelevant), the difference in the power of the TRF corresponding to the relevant and irrelevant object revealed an 10-Hz response that was initially negative and then showed a trend for an increase after 0.2 s. The trend for this increase was termed the alpha rebound by the authors. As the authors point out, the alpha rebound is best explained by considering the 10-Hz alpha activity to reflect functional inhibition of neural processing [15,17,18]. As such, the initial decrease of about 10 Hz power in the difference-TRF may reflect a selective release from inhibition associated with the relevant object, followed by a later increase in active inhibition of the same object. This finding held up even when the task required participants to track the objects while the objects were moving. This shows the sampling mechanism is not based on spatial locations but is object-specific. The 10-Hz envelope of the difference-TRF thus appears to track the time course of attentional deployment between two different objects independently of their respective locations.

A second important finding was that when one object was more relevant than the other (e.g., more likely to contain a target than the other, indicated by a 75% valid attentional cue), the alpha rebound at 200–400 ms became more pronounced. Specifically, alpha power of the difference-TRF was initially negative but clearly changed sign after 200 ms. This pattern indicates that while attention is initially allocated to the most relevant object, resources are subsequently allocated to the less relevant object a few hundred milliseconds later. Importantly, the strength of the alpha rebound predicted behavioral markers of attentional deployment across individuals. Together, these findings were interpreted to reflect a sequential allocation of attention that is flexibly adjusted to changes in task context by first allocating attention to the most relevant object and a little later to the less relevant object.

Finally, something remarkable occurred when both objects were equally relevant (see Fig 4A and 4B in Jia et al.). The difference-TRF now revealed a slow oscillatory modulation of the 10-Hz envelope, such that the decrease and increase in TRF alpha power continued to alternate for nearly a second (see also Fig 1C). The authors interpret this pattern to reflect attention shifting back and forth between the two visual objects approximately every 0.5 s. As such, the allocation of attention in the presence of multiple objects appears not only to be sequential but also rhythmic in nature.

## Additional evidence for sequential sampling

The results of Jia et al. relate to other recent intriguing findings of rhythmic attentional sampling. For instance, by quantifying the detection accuracy of subtle changes in two different objects as a function of time, Landau et al. recently demonstrated that visual sampling fluctuated at a 4-Hz rhythm [19]. This is consistent with similar findings by Fiebelkorn et al., who, in addition, investigated the spatiotemporal dynamics of between- versus within-object attentional sampling and found that attention switches between objects at a rate of about 4 Hz and within objects at about 8 Hz [20]. Furthermore, magnetoencephalographic (MEG) recordings during an object-based attention task demonstrated that the shift in visual sampling was reflected by modulations in posterior gamma-band activity [21]. This is important, as neuronal activity in the gamma band has been proposed to reflect feed-forward processing [22,23]. Another recent study by Song et al. suggests that sequential attentional sampling is mediated through oscillations in the 3–5-Hz theta band [24]. Together, these studies provide converging evidence for the notion that attentional sampling is indeed sequential and “jumps” from one object to another. It should be pointed out, however, that the sampling frequency identified by Jia et al. was about 2 Hz (about 2 “samples” of each object per second; see Fig 1C), which is slower than the approximately 4 Hz identified in previous research. As we will discuss below, the factors determining the sequential sampling frequency need to be elucidated in future research.

## Future questions

The findings by Jia et al. raise several questions that deserve attention in the future (Fig 1D). One core question pertains to what happens in natural settings in which more than two objects are shown. One might hypothesize that attention will then jump sequentially among all the objects, potentially resulting in less frequent sampling of individual objects when attending multiple objects [25]. As such, the sequential mechanism might relate to the work on visual search by Treisman et al. [26–28]. The scan rate implied by the Jia et al. study is, however, quite slow compared to the scan rate of the Treisman studies, possibly due to differences in the precise detection task and/or the number of items that were used across studies. The relationship between the physiological mechanism identified by Jia et al. and the classical psychophysical work on sequential scanning, which has thus far remained largely unconnected to findings of rhythmic sampling, deserves to be uncovered.

A related question pertains to the factors that determine the sequential allocation of attentional resources across different objects or locations. As Jia et al. demonstrate, the relative task relevance of different objects is an important determinant of the pattern of attentional sampling, suggesting that sequential sampling may be under top-down control. Previous research has shown that reward paring and long-term memory but also visual saliency modulate the allocation of attention [29,30]. How do these factors modulate the spatiotemporal dynamics of sequential sampling? The approach presented by Jia et al. now provides a tool to address these questions.

A third outstanding question is how rhythms of attentional sampling relate to naturally occurring neuronal oscillations. For instance, occipital activity in humans and monkeys shows strong neuronal oscillations in the theta (5–8 Hz) and alpha (8–13 Hz) bands [31–35]. How do these rhythms link to the observed sequential sampling that also is rhythmic in nature? It is important to uncover if and how these different endogenous rhythms are related.

A final question concerns the temporal organization of visual exploration and search. It is plausible that the sequential attentional sampling process is part of the mechanism informing the visual system of where to saccade next. Saccades are obviously a major factor in the allocation of attention [36–38]. By saccading, we shift our gaze to different parts of a visual scene 3–4 times per second [38,39]. How does sequential sampling relate to the timing of saccades? In conclusion, the findings reported by Jia et al. raise exciting questions concerning the nature, flexibility, and function of the spatiotemporal dynamics of attention.

## Abbreviations

EEG: electroencephalogram
MEG: magnetoencephalographic
TRF: temporal response function
